# Supplementary material for: Physical Activity Interventions and Their Effects on Cognitive Function in People with Dementia: A Systematic Review and Meta-Analysis
Source: Int J Environ Res Public Health. 2021 Aug 19;18(16):8753. doi: 10.3390/ijerph18168753 (PMC8394441; doi:10.3390/ijerph18168753)
Supplement: Supplementary file 1 [file ijerph-18-08753-s001.zip › Additional file 1_ search strategy.pdf]

Additional file 1- PubMed full electronic search

(((((Elderly adults [MeSH Terms]) AND ((((((((Physical activity) OR exercise training [MeSH Terms]) OR training*) OR physical exercise [MeSHTerms])) OR (((((((exercise therapy OR physical activity therapy))) OR ((physical fitness") OR (Sports))) OR acute exercise) OR isometric exercise) OR strength exercise) AND interventions)) OR ((((aerobic exercise) OR endurance*) OR cardio) OR cardiovascular))) AND ((((((((((dementia [MeSH Terms]) OR neurocognitive disorder) OR nervous system disease*) OR brain disease) OR neurodegenerative disease) OR Alzheimer's) OR ((Alzheimer's disease) OR vascular dementia)) OR (((frontotemporal) AND dementia AND disease))) OR Lewy body disease) AND (((((cognition as topic[MeSH Terms]) OR cognitive function as topic[MeSH Terms])) OR mental process) OR cognitive reserve))) AND (((((clinical trials as topic[MeSH Terms]) OR controlled clinical trials as topic[MeSH Terms])) OR controlled clinical trial[Publication Type]) OR randomized controlled trial[Publication Type]))) AND ("2000/01/01"[Date -Publication]) AND ("English"[language]).
