# Supplementary material for: Physical Activity Interventions and Their Effects on Cognitive Function in People with Dementia: A Systematic Review and Meta-Analysis
Source: Int J Environ Res Public Health. 2021 Aug 19;18(16):8753. doi: 10.3390/ijerph18168753 (PMC8394441; doi:10.3390/ijerph18168753)
Supplement: Supplementary file 1 [file ijerph-18-08753-s001.zip › Additional file 2_ methodological quality.pdf]

Additional file 2– Parameters for the methodological quality assessment

Previous reviews recommendations on methodological aspects [1-4].

- Comprehensive cognitive measures: studies should include comprehensive cognitive measures that assess not only global cognition but other cognitive subdomains [2].
- Measurements throughout the intervention period: studies should conduct more than one measure during intervention time. Taking into account that dementia is a progressive disease, different measurements during the intervention time, might permit to identify at which point PA have a greater effect by delaying the cognitive impairment [2].
- Long-term follow up: Studies should conduct more than one long-term follow up; since using, a follow-up evaluation might contribute to identify whether the effects were maintained over time [1-3].
- Target dementia type: studies should targeted and assessed a specific type of dementia. Separating subjects with mild, moderate, and severe dementia, will enable to identify if all stages of the disease benefit equally [4].
- Target dementia stage: studies should target dementia stages (mild, moderate, sevre); to identify in which particular stage the person can be receive greater benefits from physical activity [4].
- Provide standardized information for PA characteristics: studies should provide clear information that describes PA type (cardiovascular, strengthening, combined with cognitive training); duration (minutes per session and total intervention length); frequency (times a weak); and intensity (reported PA intensity and displayed % of the maximum heart rate, Borg Rating of Perceived Exertion) [3, 4].

References

1. Du, Z., et al., *Physical activity can improve cognition in patients with Alzheimer's disease: a systematic review and meta-analysis of randomized controlled trials.* Clin Interv Aging, 2018. **13**: p. 1593-1603.
2. Farina, N., J. Rusted, and N. Tabet, *The effect of exercise interventions on cognitive outcome in Alzheimer's disease: a systematic review.* Int Psychogeriatr, 2014. **26**(1): p. 9-18.
3. Jia, R.X., et al., *Effects of physical activity and exercise on the cognitive function of patients with Alzheimer disease: a meta-analysis.* BMC Geriatr, 2019. **19**(1): p. 181.
4. Ohman, H., et al., *Effect of physical exercise on cognitive performance in older adults with mild cognitive impairment or dementia: a systematic review.* Dement Geriatr Cogn Disord, 2014. **38**(5-6): p. 347-65.
