# Supplementary material for: Physical Activity Interventions and Their Effects on Cognitive Function in People with Dementia: A Systematic Review and Meta-Analysis
Source: Int J Environ Res Public Health. 2021 Aug 19;18(16):8753. doi: 10.3390/ijerph18168753 (PMC8394441; doi:10.3390/ijerph18168753)
Supplement: Supplementary file 1 [file ijerph-18-08753-s001.zip › Additional file 3 _Measurement tools.pdf]

Additional file 2 – Cognitive domains measurement tools

Cognitive domain	Test	Authors
**General intellectual functioning**<br>**Global cognitive ability**<br>**Cognitive functioning**<br><br><br><br>**Multi-domain cognitive tests**	Mini-Mental State Examination (MMSE) (1)	Karssemeijer et al., (2019); Huang et al., (2018); Henskens et al., (2018); Toots et al., (2017); Öhman et al., (2016); Kim et al., (2016); Cancela et al., (2016); Hoffmann et al., (2015); Bossers et al., (2015); Yang et al., (2015); Cheng et al., (2014); Vreugdenhil, et al., (2012); Venturelli, et al., (2011); Thurm, et al., (2011); Steinberg et al., (2009); Miu et al., (2008); Kwak el at., (2008); Christofoletti et al., (2008); Stevens & Killeen, (2006); Van de Winckel, et al., (2004)
	Clinical Dementia Rating (CDR) Scale	Öhman et al., (2016); Miu et al., (2008); Christofoletti et al., (2008)
	Alzheimer's Disease Assessment Scale (ADAS-COG)	Lamb et al., (2018); Toots et al., (2017); Kim et al., (2016); Hoffmann et al., (2015); Yang et al., (2015); Vreugdenhil, et al., (2012); Thurm, et al., (2011); Van de Winckel, et al., (2004)
	Wechsler Adult Intelligence Scale (WAIS)	Henskens et al., (2018); Lamb et al., (2018)
	Montreal Cognitive Assessment (MoCA)	Huang et al., (2018)
	WHO-University of California Los Angeles-Auditory Verbal Learning test (WHO-UCLA-AVLT)	Huang et al., (2018)
	Severe Impairment Battery-Short From (SIB-S)	Henskens et al., (2018);
	Rapid Evaluation of Cognitive Function (French ERCF)	Kemoun et al (2010)
	Groninger Intelligentie Test (GIT)	Henskens et al., (2018)
	The Stroop Color and Word Test (SCWT)	Karssemeijer et al., (2019); Hoffmann et al., (2015); Bossers et al., (2015)
**Memory**	Wechsler Memory Scale Revised (WMS-R)	Bossers et al., (2015)

	Le Rivermead Behavioural Memory Test (RBMT)	Eggermont, et al., (2009)
	Fuld Object Memory Evaluation (FOME)	Cancela et al., (2016);
		Steinberg et al., (2009)
	Hopkins Verbal Learning Test (HVLT)	
	Location Learning Test—Revised (LLT-R)	Karssemeijer et al., (2019)
**Attention and concentration**	Symbol Digit Modalities Test (SDMT),	Hoffmann et al., (2015);
	Trail Making Test (TMT)	Karssemeijer et al., (2019); Huang et al., (2018);
**Language**	Boston Naming Test (BNT), Verbal	Steinberg et al., (2009);
	fluency (VF) Test	Toots et al., (2017); Hoffmann et al., (2015);
**Visuo-spatial abilities**	Clock-Drawing test (CDT)	Öhman et al., (2016); Kim et al., (2016); Stevens & Killeen, (2006)
**Executive functions**	Trail Making Test (TMT)	Henskens et al., (2018)
	The Frontal Assessment Battery (FAB)	Henskens et al., (2018)
